# Advanced Electrodes for High Power Li-ion Batteries

**DOI:** 10.3390/ma6031028

**Published:** 2013-03-15

**Authors:** Karim Zaghib, Alain Mauger, Henri Groult, John B. Goodenough, Christian M. Julien

**Affiliations:** 1 Energy Storage and Conversion, Hydro-Québec Research Institute, 1800 Boul. Lionel-Boulet, Varennes, Québec, J3X 1S1, Canada; 2 Institut de Minéralogie et Physique des Milieux Condensés, Université Pierre et Marie Curie, 4 place Jussieu, Paris Cedex 05, 75252, France; E-Mail: alain.mauger@impmc.jussieu.fr; 3 Physicochimie des Electrolytes, Colloïdes et Sciences Analytiques, Université Pierre et Marie Curie, 4 place Jussieu, Paris 75005, France; E-Mails: henri.groult@upmc.fr (H.G.); christian.julien@upmc.fr (C.M.J.); 4 The University of Texas at Austin, Austin, TX 78712, USA; E-Mail: jgoodenough@mail.utexas.edu

**Keywords:** positive electrode, lithium nano titanate, Li-ion batteries, encapsulation

## Abstract

While little success has been obtained over the past few years in attempts to increase the capacity of Li-ion batteries, significant improvement in the power density has been achieved, opening the route to new applications, from hybrid electric vehicles to high-power electronics and regulation of the intermittency problem of electric energy supply on smart grids. This success has been achieved not only by decreasing the size of the active particles of the electrodes to few tens of nanometers, but also by surface modification and the synthesis of new multi-composite particles. It is the aim of this work to review the different approaches that have been successful to obtain Li-ion batteries with improved high-rate performance and to discuss how these results prefigure further improvement in the near future.

## 1. Introduction

Sony Energytec commercialized the first Li-ion battery equipped with a LiCoO_2_ cathode element in 1990 [1]. This battery is still used for portable applications, but fails to be used for applications demanding more power, such as electric and hybrid vehicles, mainly for safety reasons [2,3]. Since then, other cathode elements have been proposed; they can be divided into three different families. One is the family of lamellar compounds to which LiCoO_2_ belongs, obtained by the multi-ion substitution of Co. The archetype of this family is LiNi_1/3_Mn_1/3_Co_1/3_O_2_ (LNMC) first introduced by Ohzuku’s group in 2001 as a candidate cathode material [4,5]. The role of Mn is to order the Li; Ni is the electrochemically active element. Co is still needed to avoid the antisite defect corresponding to Ni^2+^ on the Li^+^ site [6] facilitated by the fact that Li^+^ and Ni^2+^ ions have almost the same ionic radius; antisite defects damage the electrochemical properties where the defect concentration exceeds 2 at. % [7].

The second family is the olivine group, the archetype of which is LiFePO_4_ (LFP) [8]. However, the low intrinsic electronic conductivity postponed its use, until the problem was solved by coating the particles with conductive carbon [9], which has been a remarkably successful surface modification. A few additional years were required before the material could be freed from any impurity [10]. Since then, the material has been extensively studied. The physical and electrochemical properties as well as the surface and size effects have been reviewed recently [11,12,13,14]. For the purpose of the present work, we can summarize the state-of-the-art by saying that there are now different synthesis routes at the industrial scale that make possible the preparation of LiFePO_4_ particles of different sizes, from submicron to the nano-range depending on the use that is targeted, that are free of any impurity. We only redirect the reader to these prior reviews for more information.

On another hand, the other members of the Li*M*PO_4_ family with *M* any mixing of Mn, Ni, Fe, Co are still not yet competitive with LiFePO_4_, despite many efforts. The substitution of Fe by these other transition-metal elements aims to increase the energy density since the *M*^2+^/*M*^3+^ redox potential *vs.* Li^+^/Li increases with the atomic number: to 3.4, 4.1, 4.8 and 5.1 V for *M* = Fe, Mn, Co, Ni, respectively. The two last voltages exceed the stability window of the organic electrolytes presently available, so attention has been focused on LiMnPO_4_ (LMP) because 4.1 V is still lower than the highest occupied orbital (HOMO) of the conventional organic electrolyte and its theoretical energy density is larger (701 mWh g^−1^, against 586 mWh g^−1^ in LFP). In practice, however, the results are disappointing because its intrinsic electronic conductivity is even smaller than that of LFP [15]. Therefore, the only hope to make it competitive as a cathode element is to follow the same recipe that proved successful for LFP, *i.e.*, decrease the size of the particles to the nano-scale and coat the particles with conductive carbon. Indeed, LMP particles of size ~50 nm have been obtained by different synthesis routes: solid-state reaction in molten hydrocarbon [16], polyol synthesis [17,18], spray pyrolysis plus ball milling [19]. The best result has been obtained by Choi *et al.* who synthesized LMP nanoplates with a thickness of 50 nm that are assembled and grown into nanorods along the (010) direction in the (100) plane [20]. A capacity of 168 mAh g^−1^ at very low rate C/50 obtained in this case is close to the theoretical value and is about the same as the capacity obtained for LFP. At faster rates, however, which are essential for electric vehicles that require high power supplies, LMP still cannot compete with LFP because the carbon coating of LMP turns out to be more difficult than that of LFP. The reason is that Fe is very reactive with carbon [21,22] while Mn is not. Indeed, despite many efforts made to obtain C-LMP, the capacity remains smaller than that of LFP; it is limited to 130–140 mAh g^−1^ at C/10 rate, and such values are reached only if the particles are immersed in a huge quantity of carbon, typically 20 wt % [23] up to 30 wt % [24,25]; the carbon cannot exceed a few wt % in commercial batteries.

The third family consists of spinel compounds, the archetype being LiMn_2_O_4_. This material has been used as a cathode element in electric-car Li-ion batteries; however, it suffers from the dissolution of manganese in the electrolyte, which reduces the calendar life, especially upon use above room temperature. Moreover, LiMn_2_O_4_ suffers from a reduction of the cycling life at high C-rate. As a consequence, LFP increasingly replaces this compound. The main attention today is focused on another spinel structure, LiMn_1.5_Ni_0.5_O_4_ (LMN), which is of great interest because it provides access to the Ni(IV)–Ni(II) formal valences at about 4.7 V *vs.* Li^+^/Li [26,27]. However, cathode/electrolyte surface reactions lead to degradation in the electrochemical performance [28].

The recent works that are reviewed here have been guided by the following strategies. Now that it is possible to reduce the concentration of the antisite defects in the layered LNMC to a negligible amount, progress may come from surface modification in order to cure the disorder of the surface layers; surface disorder is responsible for an increase of the electronic and ionic resistivity. For LFP, which is already conquering the market for the new generation of hybrid and electric vehicles, progress may come from the reduction of the cost of the synthesis of nanoparticles by new synthetic methods [29], or by optimizing more expensive synthesis routes such as the hydrothermal process that make possible the tuning of the shape of the particles (see [30] and references therein). To improve the energy density of cathode elements in the near future, however, the efforts have been targeted to increasing the electrochemical properties of the LMP and LMN particles that are penalized by surface problems, *i.e.*, by carbon coating in the first case, by eliminating the solid-electrolyte interface (SEI) phase in the second case. A solution that has been found is first to coat the particles with a protective layer of LFP, to take advantage of the remarkable stability of this material, and then to coat the particles with a layer of conductive carbon, taking advantage of the Fe–C affinity.

The anode of the commercialized Li-ion batteries with high power is graphite because of its low potential with respect to Li^+^/Li and high energy density with the formation of LiC_6_. However, a graphite anode limits not only the rate of charge, but also the discharge power density. The graphite layers are fragile and the insertion of lithium between the layers results in an increase of the lattice volume, which may partially break the particles and alter the SEI passivation layer. For instance, Garcia *et al.* [31] have shown that large stresses develop at high-rate discharge and contribute to the mechanic failure of the graphite electrode. The fracture of the carbon particles [32] is expected to occur above 6C rate [33]. In addition, at a high charging rate, Li plating on the graphite electrode is inevitable, leading to thermal instability, capacity fade and cell failure [34,35,36,37]. Therefore, efforts have been made to replace graphite with Li_4_Ti_5_O_12_ (LTO). This lithium titanate with a spinel structure exhibits a stable operating voltage of 1.5 V *vs.* lithium [38,39,40], which is within the commercial-electrolyte window. Moreover, the dilatation of the lattice of LTO upon lithiation is almost zero; the stress during cycling is also negligible, so that no crack of the particles is expected upon cycling. The absence of resistive SEI layer is another advantage that makes this material avoid all the factors mentioned above that limit the power density with a graphite electrode. The capacity is much smaller than that of graphite, but since it is about the same as that of the cathode elements, this limitation is not severe. The main drawback comes from the energy density because the operating voltage of the cell is decreased by the 1.5 V with LTO *vs.* Li^+^/Li. This feature is prohibitive for some applications like fully electric vehicles, for instance, but not for applications that demand more power than energy, such as hybrid vehicles. The recent results obtained on LFP//LTO cells are very promising and are also reviewed hereunder. The last part of this paper is devoted to a discussion of expectations for the improvement in the performance of Li-ion batteries that can be expected in the near future.

## 2. Surface Modification

The coating of LiFePO_4_ with carbon is a remarkable example of surface modification. The resulting effect was not detected immediately since the carbon coating was intended to put at the surface of the particles a conductive layer percolating through the powder to transport electrons between the particles and the current collector. Indeed, the first benefit is that an electron reaching the surface of the particle during the charging process can be transported to the collector via the percolating carbon matrix, limiting the slow electron path inside LFP to particles of small radius. It was later realized that the choice of the carbon precursor must be an organic compound because the carbon used for the coating also releases the hydrogen species of the organic substance to act as a reducing agent, thus avoiding the formation of Fe^3+^-based impurities [41]. Only recently, however, it was realized that the carbon deposit also modifies the surface of the particles as it cures the intrinsic disorder of the 3-nm-thick surface layer [22,23,24,25,26,27,28,29] as a result of the combined effects of Fe–C affinity and annealing; the carbon is deposited at 650–700 °C. The Fe–C affinity is fortunate since the surface structural disorder is damaging to both the electronic and ionic conductivity.

**Figure 1:**
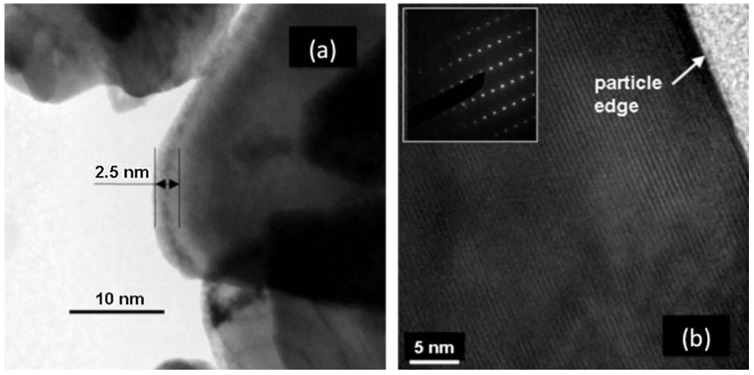
High resolution transmission electron microscopy (HRTEM) features of LiNi_1/3_Mn_1/3_Co_1/3_O_2_ (LNMC) powders for as-grown (**a**) and heat treated sample with sucrose at 600 °C for 30 min in air (**b**). (Reprinted with permission from [57]. Copyright 2011 Elsevier.)

For lamellar compounds, the electronic conductivity is not a problem. Nevertheless, the delivered capacities of these compounds fade when a high-rate current density is applied [42,43]. Aurbach *et al.* [44] have suggested that the capacity retention is strongly dependent on the surface chemistry of the particles because of resistive SEI layer. That is why many attempts have been made to protect the surface of LiCoO_2_ with metal oxides such as Al_2_O_3_, TiO_2_, ZrO_2_, MgO [45,46,47,48,49], or other compounds such as FePO_4_ [50], LiFePO_4_ [51], and Li_4_Ti_5_O_12_ [52]. In particular, the coating with ZrO_2_ improves the capacity retention of LiCoO_2_ during high-potential cycling [49]. Similar efforts have recently made for LNMC by covering the particles with several substances such as LiAlO_2_ [53] and Al_2_O_3_ [54]. The carbon coating that is so successful for LFP also seems efficient to improve the cycle life of Li-ion batteries with LNMC [55] and LiCoO_2_ as well [56]. However, the lack of surface analysis makes questionable the nature of the passivation layer and the existence of a carbon deposit. The role of carbon has been detailed only recently [57]. In the reported work [57], LNMC with an average particle size of 250 nm was first synthesized. One part of the powder was mixed with a organic substance such as sucrose and starch followed by an annealing process at moderate temperature (700 °C), a procedure that has been efficient to coat LFP particles with conductive carbon. The addition of sucrose or starch improved performance. However, in our investigation, no carbon could be detected at the surface of the LNMC particles, even by Raman spectroscopy [57], contrary to the prior work [55] that reported a nanolayer of carbon, although much less regular than on LFP. This difference presumably comes from the fact that the amount of carbon in the preparation process was only 3 wt %. The situation is somehow similar to the case of LMP case reported in the introduction, namely carbon coating requires more carbon. This is not important, however, because the electronic conductivity of LNMC is sufficiently high so that it does not limit the performance of the cathode. On the other hand, annealing in the presence of sugar has a major effect by curing the disorder of the surface layer, just like the case of LFP. This is evidenced in the Transmission Electron Microscope (TEM) images in Figure 1. Before the introduction sugar, the surface layer, about 5 nm thick, was strongly disordered, while after heating in presence of sugar, the surface layer was crystallized. Note that the as-grown sample with the disordered surface layer was prepared at temperature as high as 900 °C, while the annealing in presence of sugar was performed at 600 °C only. Therefore, the crystallization of the surface layer is not a simple thermal annealing effect, *i.e.*, the presence of sugar during the annealing process is responsible for it.

**Figure 2:**
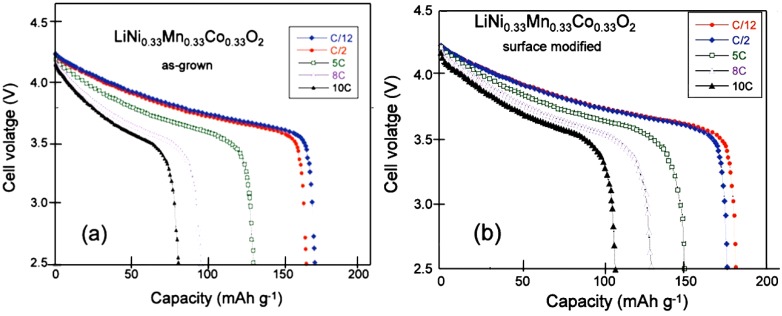
Discharge cell voltage *vs.* gravimetric capacity of the Li//LNMCO cells for various C-rate for (**a**) the as-grown cathode material and (**b**) heat treated sample with sucrose at 600 °C for 30 min in air. (Reprinted with permission from [57]. Copyright 2011 Elsevier.)

**Figure 3:**
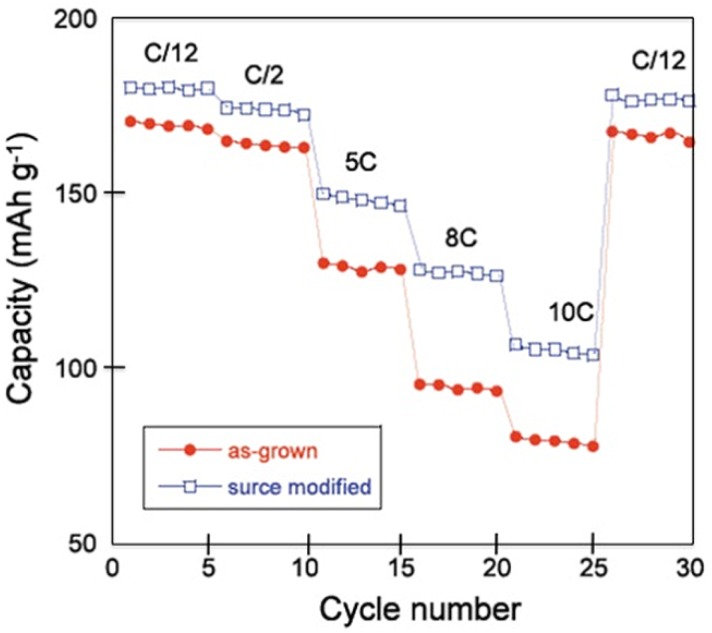
The modified Peukert plots of Li cells for the as-grown and the surface modified LNMC cathode material. (Reprinted with permission from [57]. Copyright 2011 Elsevier.)

To investigate the effect of this surface modification on the electrochemical properties, two cells were prepared with Li anode, and 1.0 mol L^−1^ LiPF_6_ in a mixture of ethylene carbonate (EC) and diethyl carbonate (DEC) (1:1, v/v) as electrolyte. The cells differ only by their negative electrode: one cell was prepared with as-grown LNMC anode, the other one with the same LNMC sample after heat treatment in presence of sugar. In addition, the same tap density was used so that a quantitative comparison between the two cells is meaningful. Figure 2a,b shows the potential *vs.* capacity curves of these cells cycled between 2.5 and 4.2 V at different C-rates. Heat-treated samples show not only improvement in the discharge capacity values, but also improvement in the capacity retention upon increasing the C-rate value. The polarization in the discharge curve of the surface-modified sample is also reduced in comparison with that of the non-treated sample. The modified Peukert plot in Figure 3 corroborates the better performance of the material after the heat treatment with sugar, the capacity of which is raised to 107 mAh g^−1^ at 10C, against 81 mAh g^−1^ before the treatment.

The improvement of the electrochemical performance due to the crystallization of the surface layer can be understood as follows. First, the electronic conductivity is affected by the structural disorder. This has been evidenced by transport experiments that have shown an increase of conductivity when the calcination temperature used in the synthesis process increases from 800 up to around 1000 °C, owing to improved crystallinity of the materials [58]. In fact, a high crystallinity is essential to obtain good electrical conductivity [58]. We can also presume that the disordered layer acts as a diffusion barrier impeding the motion of the Li^+^ ions, but impedance spectroscopy measurements still needs to be done to confirm this presumption. It should be noted that the improvement of the electrochemical properties due to the crystallization of the surface layer is quite comparable to the improvement obtained by coating LNMC with different substances [45,52]. Since, however, no surface analysis has been performed, except in few cases [46,47,51], we can even question whether the improvement is due to the coating or to a reconstruction of the surface layer. In any case, the surface modification simply obtained with table sugar is less expensive than a coating with other metal oxides or other compounds.

## 3. Multi-Composites

**Figure 4:**
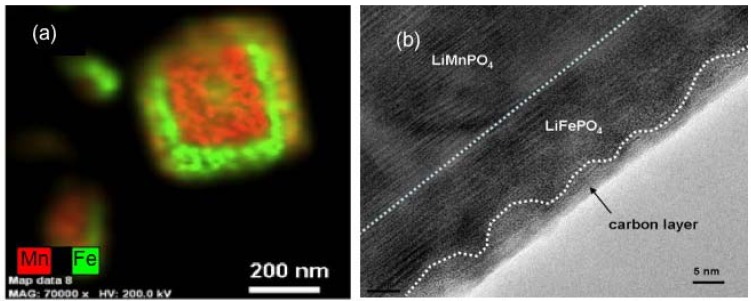
(**a**) Energy Dispersive X-ray Detector (EDX) spectra at three spots show that both Mn and Fe in the analyzed areas with Mn peak intensity higher than Fe’s one. On average, the relative concentration Mn:Fe is 2/3:1/3. (Cu and Si are from the sample support); (**b**) Transmission Electron Microscope (TEM) image showing the continuous carbon coating deposited onto the LFP layer. (Reprinted with permission from [59]. Copyright 2012 Elsevier.)

In the case of olivine compounds, a simple surface modification is not sufficient, because their low electronic conductivity requires a conductive coating of the particles. We have already pointed out in the introduction that the coating is easily done to obtain C-LFP, but more difficult in the case of LMP. One solution in this latter case consists of synthesizing the LMP particles first and coating them in a second step with a thin layer of LFP following the synthesis process described in [59]. The energy-dispersive X-ray image map in Figure 4a of Fe and Mn elements in one particle shows that this coating has been successful. It is then straightforward to coat these composite particles with carbon (third step in the synthesis process), following the same procedure as the one used for LFP. The only difference is that the sintering temperature, which is usually 650–700 °C in the case of LFP, has been decreased to 600 °C because the LMP is known to be damaged when heated at T > 650 °C. This decrease of the sintering temperature results in a carbon coat of the LFP layer that is less regular, but it is continuous as it can be seen in the TEM image in Figure 4b. In this process, we take advantage of the catalytic effect of Fe to coat the particle, since the carbon is deposited on the buffer LFP layer.

**Figure 5:**
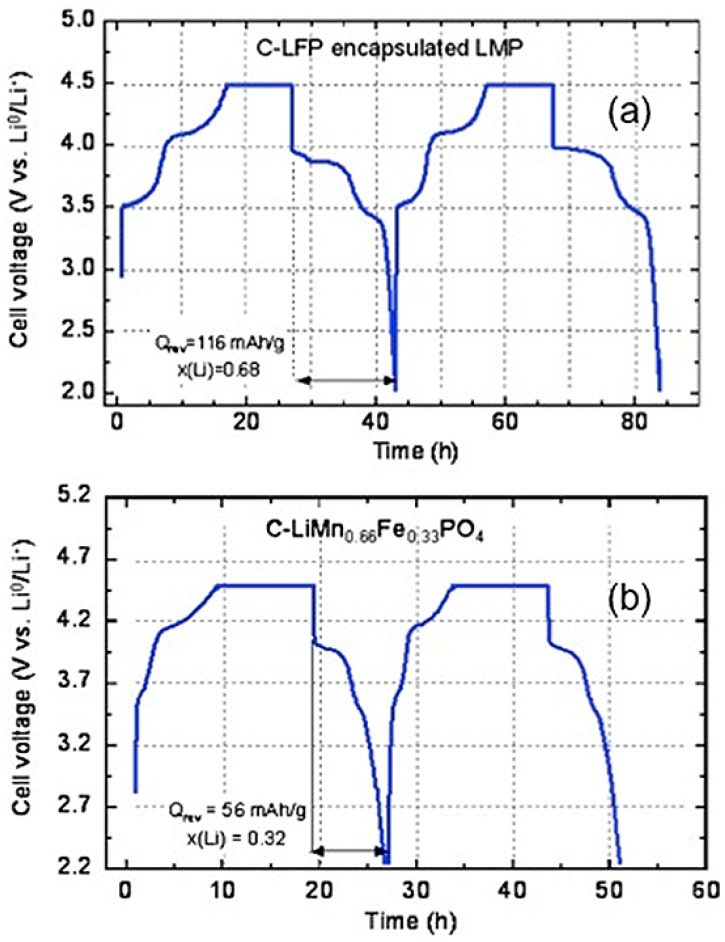
Voltage as a function of time in charge/discharge conditions corresponding to theoretical rate C/24 for the composite C-LiFePO_4_-LiMnPO_4_ and LiMn_2/3_Fe_1/3_PO_4_ for comparison. (Reprinted with permission from [59]. Copyright 2012 Elsevier.)

Moreover, the LFP layer is active and participates to the lithiation-delithiation process, so that it does not reduce the capacity. This participation is illustrated in Figure 5, which shows the variation of the potential with time for the first two cycles (same anode and same electrolyte as in the previous section). The current has been fixed to the value that would correspond to theoretical cycles at C/24 rate. The flat part corresponds to the limit of 4.5 V imposed to the potential to protect the electrolyte. The first plateau observed at 3.4 V is characteristic of the Fe^2+^/Fe^3+^ redox potential *vs.* Li in LFP. The next plateau at 4.0 V is characteristic of the Mn^2+^/Mn^3+^ potential in LMP, so that both components efficiently contribute to the electrochemical properties.

**Figure 6:**
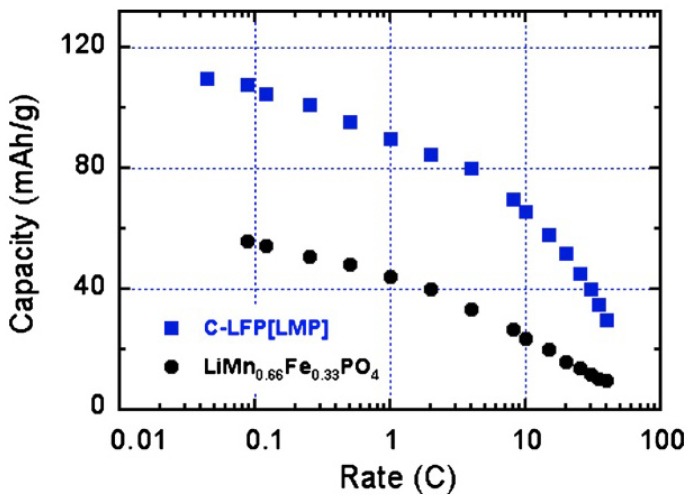
Modified Peukert plot for the C-LiFePO_4_-LiMnPO_4_ and LiMn_2/3_Fe_1/3_PO_4_ samples for comparison. (Reprinted with permission from [59]. Copyright 2012 Elsevier.)

The Mn:Fe ratio for the multi-composite particles used to obtain the results displayed in Figure 4 and Figure 5 is 2:1. The pertinence of synthesis of the multi-composite can thus be estimated by comparison of its electrochemical performance relative to that of the multi-ion solid solution with the same Mn:Fe ratio, *i.e.*, LiMn_2/3_Fe_1/3_PO_4_. This solid-solution sample has been prepared following the same procedure to make sure that the size distribution of the particles is the same as in the composite samples. Since the addition of carbon among the precursors to obtain carbon-coated samples in a one-step synthesis process limits the growth of the particles during synthesis, thus reducing the size of the particles, the LiMn_2/3_Fe_1/3_PO_4_ have been prepared first, following the same synthesis process used to obtain the LiMnPO_4_ particles in the first synthesis step, and the carbon-coat of these particles is added only in a second synthesis step [59]. The analysis by SEM and TEM experiments have confirmed that the size distribution of the C-LiMn_2/3_Fe_1/3_PO_4_ particles thus obtained is the same as that of the multi-composite C-LiFePO_4_-LiMnPO_4_ particles. The drawback is that the size of the particles is in the range 100–200 nm, while the optimum size would be smaller than 100 nm to obtain capacities close to theoretical; but in this first study, we preferred a comparison between the multi-ionic synthesis route (LiMn_2/3_Fe_1/3_PO_4_) and the multi-composite route (C-LiFePO_4_-LiMnPO_4_). The results obtained for LiMn_2/3_Fe_1/3_PO_4_ are also reported in Figure 5; they show that the multi-composite sample gives a definitely better performance. This improvement holds at any C-rate as can be seen in Figure 6. Moreover, the multi-composite sample does not age upon cycling at the scale of 100 cycles explored in [59]. The synthesis of multi-composite systems is thus a promising route to increase the operating voltage to 4 V.

**Figure 7:**
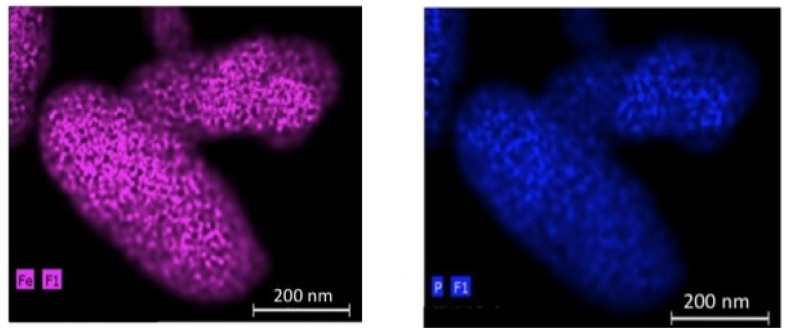
EDX maps of the P (**a**) and Fe (**b**) elements for the C-LiFePO_4_ coated LiMn_1.5_Ni_0.5_O_4_ multi-composite. (Reprinted with permission from [66]. Copyright 2012 Elsevier.)

To increase even more the operating voltage, Mn must be replaced by Ni. However, the LiNiPO_4_ olivine has very poor electrochemical performance, and the efficiency of the redox reaction does not exceed 45% [60], so that we have to go back to the spinel family. Indeed, LiMn_1.5_Ni_0.5_O_4_ is more promising because of the attractive plateau at around 4.7 V *vs.* lithium [61,62]. Nevertheless, the Fermi energy is below the HOMO of the carbonate electrolyte, so that a passivating SEI layer is formed to obtain the reversible Ni(IV)/Ni(II) redox reaction [63,64], leading to a degradation of the electrochemical performance [65]. In this case, the coating of the particles with LiFePO_4_ is expected to protect the surface of LiMn_1.5_Ni_0.5_O_4_ from reaction with the electrolyte. The multi-composite LiMn_1.5_Ni_0.5_O_4_ coated with C-LiFePO_4_ has been synthesized with a novel mechano-fusion dry process [66]. The EDX maps of P (a) and Fe (b) elements for the 200 nm-thick particles in Figure 7 show that the C-LiFePO_4_ coat is uniform.

**Figure 8:**
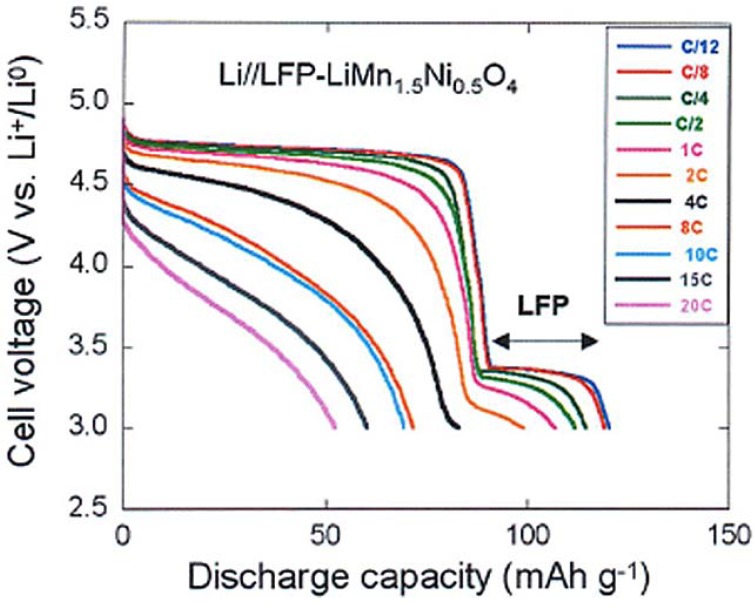
Discharge profiles of the Li//C-LiFePO_4_-coated LiMn_1.5_Ni_0.5_O_4_ cell at different C rates. (Reprinted with permission from [66]. Copyright 2012 Elsevier.)

**Figure 9:**
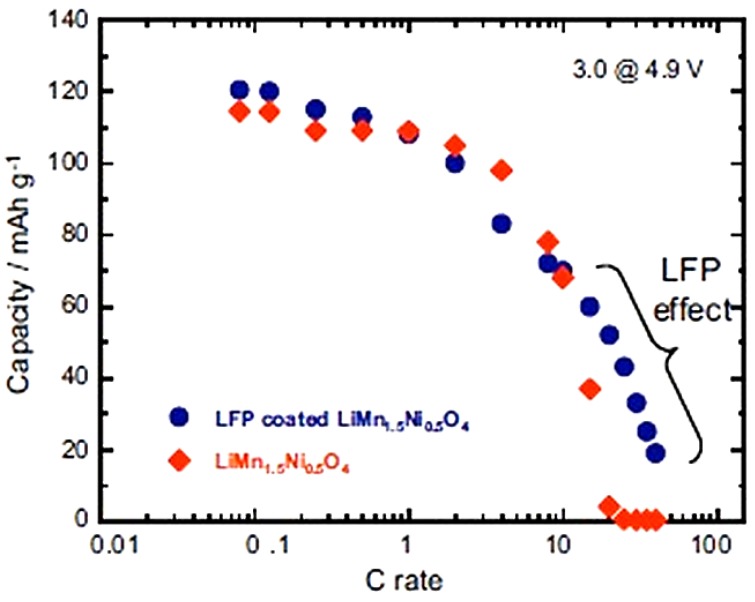
Modified Peukert plot of the Li//C-LiFePO_4_-coated and uncoated LiMn_1.5_Ni_0.5_O_4_ cells between 3.0 and 4.9 V. (Reprinted with permission from [66]. Copyright 2012 Elsevier.)

To test the electrochemical performance, a cell has been prepared with the same Li-anode and same electrolyte as in the prior sections. The cell has been cycled in the voltage range 3–4.9 V. At slow C-rate, the voltage profile in Figure 8 shows the plateau at 3.4 V characteristic of the LiFePO_4_ part, followed at higher potential by the contribution of the spinel part. As in the previous case, we have compared the electrochemical performance of the bare LiMn_1.5_Ni_0.5_O_4_ particles with that of the same particles after coating with C-LiFePO_4_. As a result, taking into account that the fraction of the spinel part with respect to the olivine part in the multi-composite powder is in the ration 1/0.8, we find that the capacity up to 10C in Figure 8 is just the sum of the spinel part and the olivine part, as expected. At higher C-rate, however, the capacity is much higher, as can be seen in the modified Peukert plot in Figure 9.

**Figure 10:**
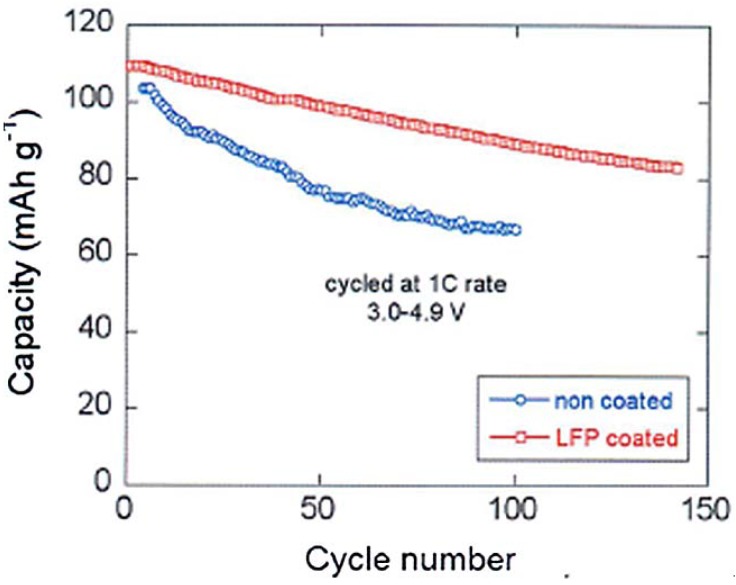
Cyclability of the Li/LiMn_1.5_Ni_0.5_O_4_ and Li/LiFePO_4_-coated LiMn_1.5_Ni_0.5_O_4_ cells at 1C between 3 and 4.9 V. (Reprinted with permission from [66]. Copyright 2012 Elsevier.)

With respect to the capacity of uncoated LMN, the analysis [66] shows that the capacity of the spinel part has increased by 60% already at 15 C and has been multiplied by the factor 13.3 at 20C after coating. We attribute this effect to the protection of the C-LiFePO_4_ coat that has isolated the spinel part from the electrolyte, thus preventing the formation of a resistive SEI layer responsible for the poor performance of the bare LMN particles. This hypothesis is confirmed by the major improvement of the capacity retention upon cycling reported in Figure 10. Note that a simple improvement of the electronic conductivity of the multi-composite powder by a carbon coat of LFP may also contribute to an improvement of the capacity at high C-rate, but it cannot have any effect on the aging upon cycling. Figure 10 is thus the best evidence of the protective effect of the C-LFP against the reaction of the spinel with the electrolyte.

To increase even more the operational voltage, and to switch to the 5 V-class of electrode materials, one has to consider cobalt compounds. Actually, it is possible to return to the olivine family because, while the electrochemical properties of LiNiPO_4_ are very poor as already mentioned, it is possible to lithiate and delithiate LiCoPO_4_ even though its capacity retention upon cycling is poor. Coating of LiCoPO_4_ by a nano-film of LiFePO_4_ has been obtained by a sol-gel process [67]. A similar improvement of the capacity retention upon cycling as the one reported in Figure 10 has been obtained in this case (see Figure 3 in [67]), which is again attributable to the protection of the LFP coat. Note that the LFP layer was not carbon-coated in this case, at least intentionally. It is possible that the particles have been unintentionally carbon-coated owing to the carbon present among the precursors, but it is impossible to know it since no detail on the choice of the precursors have been given in [67] and no characterization of the surface (by Raman spectroscopy for instance) has been done to check the presence of carbon, which is needed to optimize the electrochemical properties. In any case, coating the lamellar, spinel, and olivine compounds by C-LFP is always beneficial to the performance of the cathode elements; it improves the capacity, in particular at high C-rate, and protects the core particles from chemical reaction with the electrolyte.

## 4. The Li_4_Ti_5_O_12_ Negative Electrode

**Figure 11:**
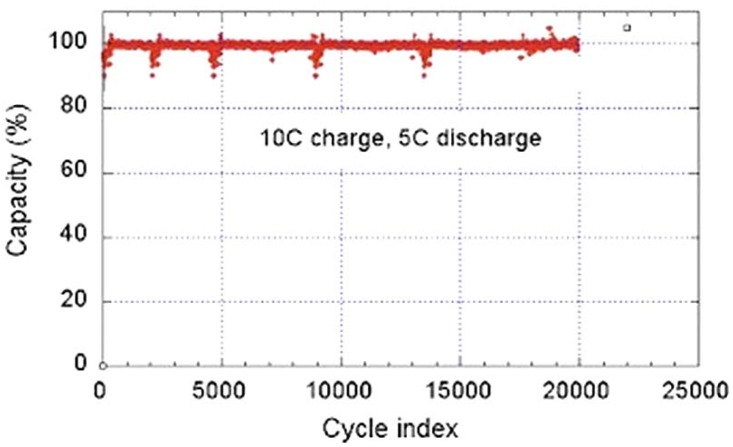
Cycle life of a LiFePO_4_/EC-DEC-1*M* LiPF_6_/Li_4_Ti_5_O_12_ 18650-cell. The cycle charge rate is 10C (6 min) at 100% state of charge, the discharge rate is 5C (12 min) at 100% depth of discharge during the test (Reprinted with permission from [68]. Copyright 2011 Elsevier).

**Figure 12:**
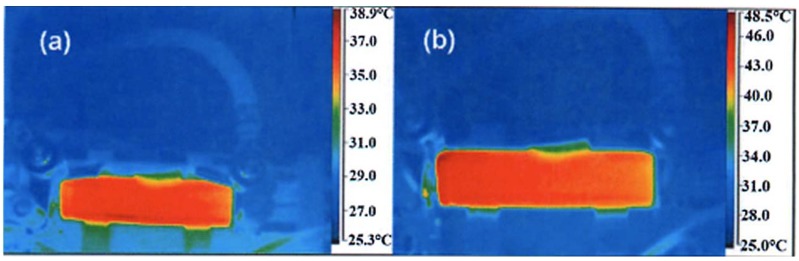
Thermal infrared image of the LTO//LFP “18650”-type cell recorded during charge at rate 50C, at full charge 2.1 V (bottom), and total discharge at 1.0 V (top). The electrolyte is with 1 mol L^−1^ LiPF_6_ in EC-DEC (1:1). (Reprinted with permission from [68]. Copyright 2011 Elsevier.)

We have already mentioned in the introduction that the graphitic negative electrode is responsible for the aging (both calendar life and cycling life) of the battery and also for the limitation on charging rate and the discharge power of the LFP-based batteries. That is why efforts have been made recently to get rid of the graphite and replace it by LTO. This spinel material has several properties that have promoted it as an anode element. First, the change of volume upon lithiation and delithiation is negligible, thus avoiding the fatigue effects of the graphite particles. The absence of any resistive SEI layer is a second advantage. Third, the lithiation in LTO is also a two-phase system, allowing fast cooperative motion of the Li^+^ ions as in the iron phosphate case. Therefore, this anode avoids the Li plating that is inevitable with graphite at high C-rate. The substitution of the graphite by Li_4_Ti_5_O_12_ has led recently to C-LFP//LTO batteries with excellent performance [68]. While the graphite anode limits the use of the battery typically to 3000 cycles and discharge rate to 6C, the C-LFP//LTO 18650-size cell delivers a capacity of 800 mAh, retains full capacity after 20,000 cycles performed at a charge rate 10C (6 min) with a discharge rate of 5C (12 min), and retains 95% capacity after 30,000 cycles at a charge rate 15C (4 mn) and a discharge rate 5C at 100% depth of discharge (DOD) and 100% state of charge (SOC) [68]. This performance is illustrated in Figure 11. Even better performances have been achieved by coating the LTO particles with carbon. The C-LFP//C-LTO 18650-size cells formed with particles of average size 90 nm for both electrodes retains more than 80% of rated capacity at 60C charge rate [69]. In addition, the temperature at any place inside the cell, measured by infrared camera, remains low. As an example, the thermal infrared images obtained at full charge and full discharge at a rate of 50C are reported in Figure 12. These images show that the maximum temperature reached under such conditions does not exceed 48.5 °C at full charge, and 38.9 °C at full discharge. These results have been obtained with the commonly used electrolyte 1 mol L^−1^ LiPF_6_ in ethylene and diethyl carbonates EC-DEC (1:1). This electrolyte, however, is sensitive to a raise of temperature. First, as T increases, LiPF_6_ degrades in the presence of organic solvents; the product of this decomposition, HF, induces acid dissolution of cathode-active materials [70,71]. Second, the organic solvents can decompose the SEI layer [72,73] and generate direct reactions between cathode-active materials and the electrolyte [74]. The consequence is a fast deterioration of the batteries at high temperature, and, in practice, is the reason why the electric cars must be equipped with a cooling system that keeps the temperature of the Li-ion batteries below 30 °C to avoid too fast aging. However, the substitution of graphite by LTO makes possible the use of other electrolytes and salts, more stable at higher temperature. The potential of the LFP//LTO is 1.9 V. This low voltage reduces the energy density with respect to the 3.4 V obtained with graphite. However it has another advantage: it is small enough so that LiPF_6_ can be replaced by the much more stable lithium bis(trifluoromethanesulfonyl)imide (LiN(CF_3_SO_2_)_2_, LiTFSI) salt without inducing corrosion on the aluminum collector. In particular, a major improvement has been achieved at any C-rate with the 0.5 mol L^−1^ LiTFSI + 1 mol L^−1^ LiBF_4_ in EC–γ-butyrolactone (GBL). At 60 °C, the discharge capacity of a 2032-size coin LFP//LTO cell with this electrolyte remains larger than 120 mAh g^−1^ up to rate 10C. At 40C rate, the capacity remains as high as 92 mAh g^−1^, while it drops to 20 mAh g^−1^ with LiPF_6_ in EC-DEC owing to the strong degradation of LiPF_6_ above 30 °C. Therefore, this combination LFP with LTO can be used to increase significantly the operating temperature range of the battery.

## 5. Discussion

The fact that the multi-composite C-LiFePO_4_//LiMnPO_4_ has better electrochemical properties at any rate than LiMn*_y_*Fe_1−*y*_PO_4_ with the same proportion of [Mn]/[Fe] illustrates the fact that the recent synthesis route of multi-composite cathode elements is more promising that the more classical attempt to synthesize multi-ion particles. To understand this effect, we note that the Jahn-Teller (JT) distortion associated to the presence of Mn^3+^ may induce Mn^3+^-rich inhomogeneities in the solid solution that distort the lattice locally to increase significantly the strain field evidenced in [75]. The resulting structural disorder may reduce the diffusivity of the Li^+^ ions and that of the electrons to decrease the electrochemical performance. For the same reason, the structural disorder of the surface layer reduces the electrochemical performance of the lamellar compounds and of the olivine family as well. A second reason for the degradation of the electrochemical performance comes from the fact that the surface of the olivine is disordered in the absence of a carbon coat, which increases the resistance to Li^+^ transfer across the electrolyte interface. A third reason has not been pointed out so far; the Fe^3+^ ions generated by delithiation of the surface layer upon exposure of the particles to moisture are in the high-spin state where the surface layer is well-crystallized, but in the low spin state where the surface layer is disordered (*i.e*., for uncoated LiFePO_4_ particles). Since Fe^3+^ in the low-spin state is significantly smaller, it will contribute to the structural disorder and thus to the degradation of the electrochemical properties. The thickness of the disordered surface layer (DSL) of as-grown, non-coated particles of both lamellar and olivine materials is small, typically 3 nm, but the disorder inside this layer is very large so that it has sizeable effects, as shown in this review. It is, therefore, important to modify the surface layer so that it is well-crystallized, which can be done by heat treatment in presence of an organic compound.

The poor performance of multi-ion cathode elements is not limited to the case of LiMn*_y_*Fe_1–*y*_PO_4_; it extends to the other Li*M*PO_4_ members of the olivine family where *M* is a mixture of Fe with other transition-metal ions. This is true not only for LiNi*_y_*Fe_1–*y*_PO_4_ [76], which is not surprising since low-spin Ni^3+^ is also a strong JT ion, but also for LiCo*_y_*Fe_1–*y*_PO_4_ [77]. In both cases, a partial substitution of Fe results in a decrease of the capacity and a poorer capacity retention. It is then not surprising that the same degradation in the performance is observed in presence of three *M* elements [78,79,80], or even four *M* elements [81] obtained by mixing Fe, Mn, Ni and Co ions. Indeed, one reason for the remarkable performance of LiFePO_4_ may also be due to the fact that the Fe^2+^ ion is not a strong Jahn-teller ion, and the Fe^3+^ ion in the delithiated material is not a Jahn-teller ion because it is everywhere in the high-spin state, including the surface if the particles are carbon-coated. Indeed, while we have shown in the previous sections that C-LiFePO_4_ can deliver more than 160 mAh g^−1^ at low C-rate, and can be charged/discharged at 40C rate without aging over 20,000 cycles, no sample with Fe partly substituted by one or more transition element has ever been able to approach this performance. For these different reasons, we believe that the new route to multi-composite systems is much more promising than the multi-ion synthesis.

If the LiFePO_4_-coating of LiMnPO_4_ has been successful, it will also be *a fortiori* for LiFePO_4_-coating of LiMn*_y_*Fe_1–*y*_PO_4_ for any composition *y* of the solid solution. Actually, the recipe has already been used for *y* = 0.88 with results similar to expectation [82]. This composition, however, should not be the best, since the best electrochemical performance on these alloys has been obtained for *y* < 0.8. The whole series of LiFePO_4_-coated LiMn*_y_*Fe_1–*y*_PO_4_ has yet to be explored in the most promising range of compositions 0.5 < *y* < 0.8. The LiFePO_4_ coating also works successfully on other members of the family, such as LiCoPO_4_ [67]. LiFePO_4_ can be used to coat other cathode particles that do not belong to the olivine family. Such a coating led to a remarkable improvement of the electrochemical performance of lamellar compounds like LiCoO_2_ [51] and LiNi_0.5_Co_0.2_Mn_0.3_O_2_ [83], and more recently of the spinel LiMn_1.5_Ni_0.5_O_4_ [66]. The improvement of the electrochemical properties is linked to the remarkable stability of LiFePO_4_, which supports high voltage up to 5 V even though the Fe^2+^/Fe^3+^ potential *vs.* Li^+^/Li is 3.4 V. Moreover, the LiFePO_4_ layer participates in the electrochemical process and thus does not decrease the energy density, in contrast to other coats that have been tried in the past. Next, nano-particles of LFP can be lithiated and delithiated very rapidly [68,69], actually faster than the other compounds of the core, so that the LFP layer naturally improves the electrochemical performance at high C-rate. Moreover, as we has shown in the case of C-LFP-MN, the electrochemical performance of the LMN core at high C-rate is improved by the C-LFP coat with respect to the same LMN particles before coating. This remarkable result is because the LiFePO_4_ coat protects the core region against reaction with the electrolyte, so that any SEI layer is formed at the surface of the LFP and not at the surface of the more reactive LMN. This example, like that of LFP-coated LiCoPO_4_, gives evidence of a major step forward with the multi-composite electrodes toward the goal of obtaining operational 5 V-batteries. Another beneficial effect of the LiFePO_4_ coat comes from the fact that the oxygen pressure at equilibrium increases sharply with redox potential above 3.5 V [84]. Therefore, any attempt to increase the voltage will also increase the risk of a loss of oxygen from the cathode element. When such a loss occurs with graphite as the negative electrode, the oxygen liberated from the positive electrode comes in contact with the anodic carbon with which it will react to form CO_2_. Since this reaction is exothermic, it can generate thermal runaway of the battery. The LiFePO_4_ shell of the multi-composite particles has thus another major advantage: by preventing the oxygen from migrating outside the core region, it will insure the thermal stability of the 4 V and 5 V battery. Of course, there are still problems to be solved before we can have a safe and performing 5 V battery, like the stability of the electrolyte. However, in this area too progress is currently being made by using tetramethylene sulfone, which is stable up to 5.8 V, for instance [85,86]. Therefore, the possibility to increase the voltage of Li-ion batteries in the near future does not seem out of reach.

The energy density is not only limited by the operating voltage of the battery, but also by the fact that one Li per chemical formula can be extracted from the cathode materials that have been reviewed in the present work. Therefore, a lot of efforts are currently being made to find a new chemistry that would make possible the extraction of two Li^+^ ions instead of one. A solution that is envisioned is the replacement of the phosphorous with silicon. Two materials from a new family of transition-metal silicate, namely Li_2_MnSiO_4_ and Li_2_FeSiO_4_, have been successfully prepared and preliminary tested for positive electrode materials [87,88,89,90,91]. These materials are even more insulating than the LiFe(Mn) olivines, but again, the same solution has been found to overcome the problem, namely carbon coating, not only of the end members, but also of the solid solution Li_2_Fe_0.5_Mn_0.5_SiO_4_ [92]. If the two Li^+^ ions might be extracted reversibly, the theoretical capacity would be raised to about 333 mAh g^−1^. Unfortunately reversible extraction of two lithium ions has not been achieved owing to the structural instability of these transition-metal silicates and the subsequent amorphization that has been observed during studies of the charge/discharge operation [93,94]. To overcome this problem, the only possibility is again to decrease the size of the particles to the nano-range so that the range of the strain field is larger than the size of the particles, in which case we can hope that the lattice distortion can be accommodated not locally, but by the particle as a whole. Indeed, in a recent work [95] the authors have succeeded in preparing ultrathin nanosheets of Li_2_(Fe,Mn)SiO_4_ that has a capacity close to theoretical with a good capacity retention over 20 cycles. This is a remarkable example of the improvements that the recent nanosheet technology can bring to the field of the Li-ion batteries since the nanosheets combine two essential properties: a large surface area, which is desired to increase the surface contact with the electrolyte, and nanoscopic thickness. Of course, the stability over 20 cycles does not make the material eligible for its use in a Li-ion battery since a reversible two-lithium-ion capacity with stable cyclic performance requires a cathode that is stable toward structural and volume changes during thousands of cycles. Nevertheless, this result is remarkable if we note that it was not possible to make more than one or two cycles before. It is then a motivation to pursue the research in this field, and it illustrates that the nano-science and technology raise the hope for the increase in the energy density of Li-ion batteries in the future. Nevertheless, the structural instability mentioned for these compounds is associated to the very large variation of volume in the lithiation and the delithiation process, so that low C-rates seem mandatory to avoid cracks and deterioration of the compounds. Therefore, it is doubtful that the silicates will be able to combine high energy density and high power. We recover the same conflict that we have met with the choice between the Li_4_Ti_5_O_12_ and the graphite anode: we have to make a choice between more energy or more power.

## 6. Conclusions

Recent progress in the reduction in size of the active particles of the electrodes below 100 nm has increased the surface contact with the electrolytes, thus improving the power density of the Li-ion batteries. At the same time, however, the surface-over-volume ratio increases, so that the surface layer, as well as SEI effects, becomes increasingly important. Recent improvements for the positive electrodes have been obtained by well-crystallized surface layer of both lamellar compounds and olivine compounds. For the negative electrode, Li_4_Ti_5_O_12_ gives outstanding results when combined with the LiFePO_4_ positive electrode since this battery does not age at the scale of 20,000 cycles, even at very high C-rates. The only drawback of the substitution of graphite by Li_4_Ti_5_O_12_ is a loss of energy density.

The energy density, however, can be increased by using the remarkable thermal stability of LiFePO_4_ to coat particles working at higher operating voltage. Such multi-composite materials have improved the performance with respect to multi-ion samples of the same overall composition. Another interest of the LiFePO_4_ coating is that it protects the inner core of the particles from contact and reaction with the electrolyte, which not only prevents the formation of a resistive SEI layer, but also increases the safety of the Li-ion batteries. In particular, the multi-composite particles open the route towards the 5 V operating Li-ion batteries, and the results on the LiFePO_4_-coated LiMn_1.5_Ni_0.5_O_4_ are encouraging.

Another attempt to increase the energy density is to pursue research into new chemistry to obtain cathode elements with more than one Li-ion per unit cell. The progress in nanoscience to prepare silicates particles of few nm in size only is encouraging, but it also suggests that the Li-ion batteries will be still confronted in the near future to the choice between more energy density and more power density. The solution might be the synthesis of multi-composite cathode particles with C-LiFePO_4_ shell, which allow for more energy by increasing the operating voltage, combined with the Li_4_Ti_5_O_12_ anode that allows for much higher power densities than graphite.
